# General and Specific Strategies Used to Facilitate Locomotor Maneuvers

**DOI:** 10.1371/journal.pone.0132707

**Published:** 2015-07-13

**Authors:** Mengnan Wu, Jesse H. Matsubara, Keith E. Gordon

**Affiliations:** 1 Department of Physical Therapy and Human Movement Sciences, Northwestern University Feinberg School of Medicine, Chicago, Illinois, United States of America; 2 Research Service, Edward Hines Jr. VA Hospital, Hines, Illinois, United States of America; Texas Tech University Health Science Centers, UNITED STATES

## Abstract

People make anticipatory changes in gait patterns prior to initiating a rapid change of direction. How they prepare will change based on their knowledge of the maneuver. To investigate specific and general strategies used to facilitate locomotor maneuvers, we manipulated subjects’ ability to anticipate the direction of an upcoming lateral “lane-change” maneuver. To examine specific anticipatory adjustments, we observed the four steps immediately preceding a maneuver that subjects were instructed to perform at a known time in a known direction. We hypothesized that to facilitate a specific change of direction, subjects would proactively decrease margin of stability in the future direction of travel. Our results support this hypothesis: subjects significantly decreased lateral margin of stability by 69% on the side ipsilateral to the maneuver during only the step immediately preceding the maneuver. This gait adaptation may have improved energetic efficiency and simplified the control of the maneuver. To examine general anticipatory adjustments, we observed the two steps immediately preceding the instant when subjects received information about the direction of the maneuver. When the maneuver direction was unknown, we hypothesized that subjects would make general anticipatory adjustments that would improve their ability to actively initiate a maneuver in multiple directions. This second hypothesis was partially supported as subjects increased step width and stance phase hip flexion during these anticipatory steps. These modifications may have improved subjects’ ability to generate forces in multiple directions and maintain equilibrium during the onset and execution of the rapid maneuver. However, adapting these general anticipatory strategies likely incurred an additional energetic cost.

## Introduction

To effectively navigate our communities, we must be able to avoid obstacles, negotiate doorways, turn corners, and recover from perturbations. Executing these non-steady-state tasks require locomotor control strategies that balance the competing objectives of maneuvering (changing speed or direction) with ongoing requirements for stable gait (resistance to changes in speed or direction). Stability can be achieved via passive “self-correction” mechanisms or through active generation of corrective forces [1,2]. As passive stability increases, larger impulses will be required to overcome the body’s resistance to movement [3,4]. Highly stable locomotion patterns that position one’s body to passively resist external perturbations can limit maneuverability, because the body will indiscriminately resist any self-imposed forces intended to change one’s trajectory [5]. Alternatively, reducing passive stability could improve maneuverability because the body will be more responsive to a given volitional force [5]. For example, preemptively leaning into a turn often causes one’s center of mass (COM) to move outside of one’s base of support (BOS) [6], creating a temporary decrease in passive stability. This anticipatory adjustment reduces COM accelerations directed away from the turn [7], consequently decreasing the magnitude of self-generated impulse required to execute the maneuver. Although not well-studied in humans, the tradeoff between passive stability and maneuverability is likely present in many control strategies that could improve the ability to navigate changing environments (e.g. changing step width, joint stiffness, or trunk lean, etc.).

There are potential risks associated with decreasing passive stability to facilitate maneuvers. As stability decreases, there is an increased likelihood of falling [8] and probability that a perturbation will result in an undesirable trajectory, such as colliding with an obstacle. However, when the properties of an impending change of direction are known (initiation time and movement direction), these risks can be managed. Exposure to reduced passive stability can be limited temporally to the step(s) immediately preceding the maneuver and spatially to the direction in which one is preparing to move. Implementation of specific control strategies that facilitate a given maneuver via reductions in passive stability would be desirable when the risks of reduced stability can be managed.

In contrast, when the properties of a future maneuver are unpredictable (e.g. walking quickly through a moving crowd), adapting general control strategies that facilitate maneuverability by decreasing passive stability over many steps or in multiple directions could be undesirable because the associated risks of instability will not be limited temporally or spatially. In such cases, preparing for a variety of potential maneuvers and/or perturbations requires locomotor strategies that are both stable and maneuverable [1]. This can be achieved by selecting active rather than passive control strategies [9,10]. For example, increasing stance width will increase the ability to actively generate frontal plane movements [11] and to resist lateral perturbations [12] during standing. There is evidence that increasing step width might result in similar improvements in both stability and adaptability during gait [13]. However, improving maneuverability via active control will likely incur an additional metabolic energetic cost (e.g. increasing step width [14], increasing stance phase knee and hip flexion [15], or increasing step frequency [16]).

The simple inverted pendulum model of walking can provide insight into how and if individuals modulate passive stability to facilitate maneuverability. Based on the model, a margin of stability can be calculated as the distance between the extrapolated center of mass (XCOM)–a measure accounting for both COM position and velocity—and the base of support (BOS) [17]. When the XCOM is within the BOS, the inverted pendulum will passively self-stabilize [17]. When the XCOM position exceeds the BOS, the system is no longer passively stable and will require corrective action to remain upright. During walking, the impulse required to move the XCOM beyond the BOS will be proportional to the magnitude of the margin of stability [17]. Thus, preemptively decreasing margin of stability in the direction of an intended maneuver will facilitate the specific maneuver by decreasing the volitional impulse required to reposition the COM outside of the BOS. At the same time, this decrease in passive stability will momentarily increase susceptibility to external perturbations.

The current experiment investigated specific and general strategies used to prepare for a maneuver. We examined a rapid “lane change” maneuver involving a lateral shift in position from a straight walking path to a parallel path. In the Known condition, individuals had full information about the maneuver in advance, while in the Unknown condition the movement direction was not presented until the instance when the maneuver was to occur.

We predicted that with full knowledge of the maneuver characteristics, individuals would choose an anticipatory locomotor strategy tailored to a specific maneuver. We hypothesized that individuals would manipulate passive stability by decreasing their margin of stability in the target direction of travel during the step immediately preceding the lane change. Our results support this hypothesis: subjects significantly decreased lateral margin of stability on the side ipsilateral to the maneuver during the step immediately preceding the maneuver.

Conversely, we predicted that when individuals could not anticipate the direction of the maneuver, they would adapt general anticipatory strategies to facilitate bi-directional maneuvers. We hypothesized that during the steps prior to learning the maneuver direction, subjects would increase step width (to improve lateral force-generating ability) and step frequency (to decrease the time needed to position the appropriate foot on the ground to generate lateral forces) and adapt a crouched gait (to improve their ability to create push-off forces). This second hypothesis was partially supported as subjects increased step width and stance phase hip flexion during the anticipatory steps occurring before the maneuver direction was known.

## Materials and Methods

### Subjects

14 healthy subjects gave written informed consent and participated in the experiment. This research was approved by the Northwestern University Institutional Review Board, STU00071150. Written informed consent was obtained from all participants in the study. Subjects were 28 ± 4 years, height 1.76 ± 0.12 m, weight 73.0 ± 15.0 kg, 5 males / 9 females, and right-leg dominant. Subjects had no physical impairments limiting walking ability.

### Experimental setup

**Fig 1:**
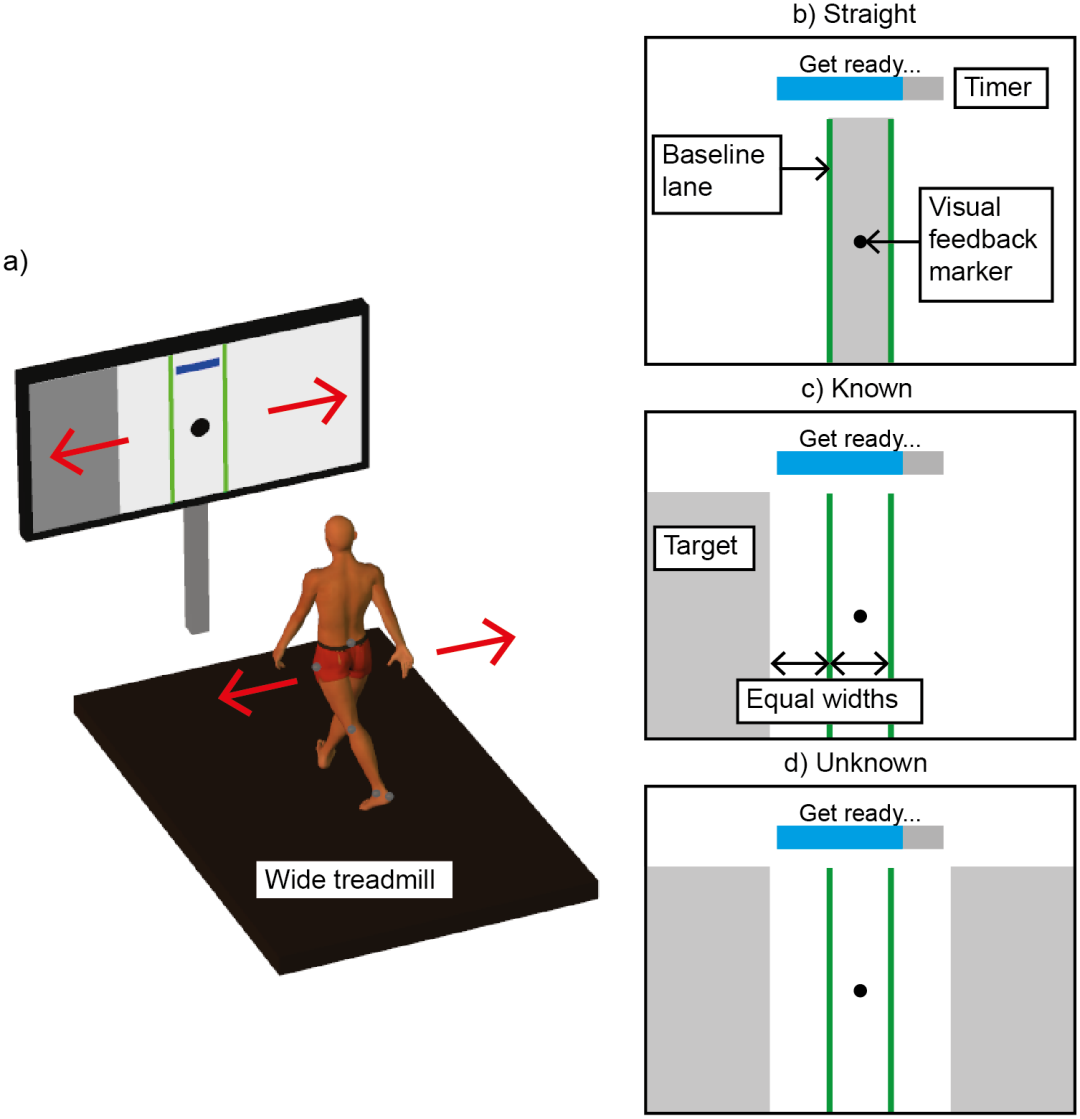
Experimental Setup. **a)** Subjects’ lateral movements (red arrows) controlled the lateral position of a real-time Visual Feedback Marker (black sphere). All trials began after subjects positioned the Visual Feedback Marker within the baseline lane shown in green. A progress bar timer then displayed a 5 second countdown and subjects were given visual instructions to “Get ready”. When time expired, the on-screen instructions changed to “GO,” and subjects maneuvered as quickly as possible to a target lane shown in grey. **b)** Straight. The target was within the baseline lane. Subjects did not maneuver. **c)** Known. A target was displayed to the either the left or right of the baseline lane during the 5 second countdown. At “GO,” subjects maneuvered to the target. **d)** Unknown. Targets were displayed simultaneously to both the left and right of the baseline lane. At “GO,” one of the targets disappeared, indicating that subjects should maneuver immediately to the remaining target.

All walking occurred on an oversized treadmill—belt width 1.39 m (Tuff Tread, Willis, TX)–providing space to comfortably side-step left or right (Fig 1a). Subjects wore a trunk harness attached to a passive overhead safety support (Aretech, Ashburn, VA). The safety system provided no bodyweight support and was adjusted to allow unrestricted travel across the treadmill.

A 60-inch monitor mounted 1.8 m in front of the treadmill provided real-time visual feedback of subjects’ lateral position. A single black visual feedback marker displayed on the monitor was calculated as the midpoint of a line drawn directly between reflective markers placed over each greater trochanter (Fig 1a).

39 reflective markers were placed on the pelvis and bilaterally on the lower limbs using a 6-DOF cluster marker setup. A 10-camera motion capture system (Qualysis, Gothenburg Sweden) recorded the 3D marker coordinates at 100 Hz and streamed data in real-time to a custom LabVIEW VI (National Instruments, Austin, TX) that created the visual display. As subjects moved left or right, the visual feedback marker moved simultaneously using 1:1 scaling. Only lateral position information was displayed to limit the cognitive demand on the subjects.

### Protocol

All walking was performed at 1.2 m/s. A fixed speed was used across subjects to constrain the spatiotemporal variables associated with preparing for and initiating the maneuver. First, subjects’ normal lateral pelvic excursion during walking was determined. On the display monitor, the visual feedback marker was overlaid with a single vertical line aligned with the treadmill center. Subjects were instructed to walk comfortably with their visual feedback marker oscillating about the vertical line. Next, we manually adjusted the width of an invisible virtual lane centered on the vertical line. The virtual lane width was gradually increased until subjects achieved 20 consecutive seconds of walking without the visual feedback marker leaving the lane. The width of this lane was multiplied by 1.5 and used as the baseline lane width for all future trials, because narrower lanes limited the ability of some pilot subjects to implement their preferred lane-change strategy. The baseline lane width used during the experiment was 0.13 ± 0.012 m for all subjects. Once the lane width was determined, subjects practiced 5 minutes of walking while maintaining the visual feedback marker within the visible baseline lane.

Next, subjects performed blocks of 20 consecutive trials for each of the following walking conditions: Straight (control steps, no maneuver), Known (maneuvers to the left or right), and Unknown (maneuvers to the left or right). The order of the blocks/conditions was randomized. All trials began with subjects walking at a steady-state speed with the visual feedback marker positioned inside the baseline lane. Subjects were given rest as needed between conditions.

#### 1. Straight

Subjects were instructed to walk with the visual feedback marker within the baseline lane for a 5-second period. A progress bar timer on the monitor provided continuous feedback on the time remaining in each trial (Fig 1b). The 5-second period displayed on the progress bar timer began after subjects had walked with their visual feedback marker within the baseline lane for a full second. During the 5-second period, visual instructions to “Get Ready” were also displayed on the monitor. At the conclusion of the 5-second period, the visual instructions changed to display the word “GO.” During the Straight trials the subject was instructed to continue walking within the baseline lane after they were given the “GO” signal. The progress bar timer and the “Get Ready” and “GO” signals were presented during the Straight condition to match the visual cues presented during the preparatory periods of the Known and Unknown walking conditions. Trials were repeated if the visual feedback marker exited the baseline lane at any time during the trial.

#### 2. Known

Subjects were instructed to walk with the visual feedback marker held within the baseline lane for a 5-second preparatory period displayed on the progress bar timer. As described for the Straight condition, the 5-second period began after subjects had walked with their visual feedback marker within the baseline lane for a full second. During this period, a target lane located either left or right of the baseline lane was displayed along with visual instructions to “Get Ready” (Fig 1c). When the preparatory period elapsed, the visual instruction changed to “GO,” and the near border of the target lane turned red, indicating that the subject should maneuver as quickly as possible to the target lane. The “GO” signal always occurred at the end of the 5-second preparatory period and was not tied to any specific gait event. The progress bar timer provided subjects with continuous feedback about the time remaining until the “GO” signal would appear. During the preparatory period subjects were free to modulate their gait pattern as desired to best respond to the “GO” signal. However, trials were repeated if the visual feedback marker exited the baseline lane at any time prior to the “GO” signal.

The distance between the nearest borders of the baseline and target lanes was equal to the baseline lane width. To minimize potential speed-accuracy tradeoffs that could influence subjects’ maneuver strategies, the target lane width was infinite, meaning subjects only had to move the visual feedback marker a threshold distance (past the near border of the target lane) to successfully perform the maneuver.

The trial concluded when subjects held the visual feedback marker in the target lane for one second. Immediately upon completion of each trial, subjects were shown their maneuver time, calculated from the “GO” signal until their visual feedback marker first entered the target lane. This feedback was used to motivate subjects to maneuver as quickly as possible. Prior to beginning this condition, subjects were given 1–3 practice trials with the treadmill off to become familiar with the task.

#### 3. Unknown

Same as Known, but target lanes were displayed simultaneously to both the left and right of the baseline lane during the 5-second preparatory period (Fig 1d). At the instant the “GO” signal was displayed, one of the target lanes disappeared, indicating that the subject should move immediately to the remaining target lane.

### Analysis

To focus on each subject’s optimal maneuver strategies, we analyzed only the 6 fastest (minimum time from “GO” to when the target lane was reached) side-step trials from the Known condition in each direction, left and right (12 Known maneuvers/subject total). For the Unknown condition, we analyzed the 6 fastest trials where the last initial foot contact prior to “GO” was on the left limb and the 6 fastest trials where the last initial foot contact was on the right (12 Unknown maneuvers/subject total). For the Straight condition, we analyzed one left and one right step from each of the last 6 trials (12 Straight steps/subject total) to compute average “control” step values for the left and right sides.

People typically turn using a side-step—pushing off the outside limb and laterally stepping the inside limb in the intended direction of movement—instead of a cross-over step—pushing off the inside limb and stepping the outside limb across the body [7]. In the current study, across all subjects and trials, 80% of the fastest Known maneuvers employed a side-step. Because the kinematics of the side-step and cross-over step are very different, it was not appropriate to group these strategies together. Thus, only the fastest side-step trials were analyzed for the Known condition. One subject took predominantly cross-over steps for the Known condition when maneuvering to the left, so fewer than 6 steps were available for analysis in this case. Step type was not considered when selecting the Unknown condition trials to analyze. This decision was based on the assumption that subjects would not demonstrate behaviors that were specific to a singular maneuver direction prior to learning the actual direction of the maneuver.

Kinematic marker data was processed using a combination of Visual3D (C-Motion, Germantown, MD) and a custom Matlab (Mathworks, Natick, MA) program. The marker data was low-pass filtered (Butterworth, cut-off frequency of 6 Hz) and gap-filled. Then the time of left and right initial foot contact (IC) and toe-off (TO) events were identified for each step based on the vertical position of the Calcaneus marker, and the fore-aft positions of the 2^nd^ and 5^th^ Metatarsal markers. Hip and knee joint angles were calculated using Visual3D’s model-based computation functions.

**Fig 2:**
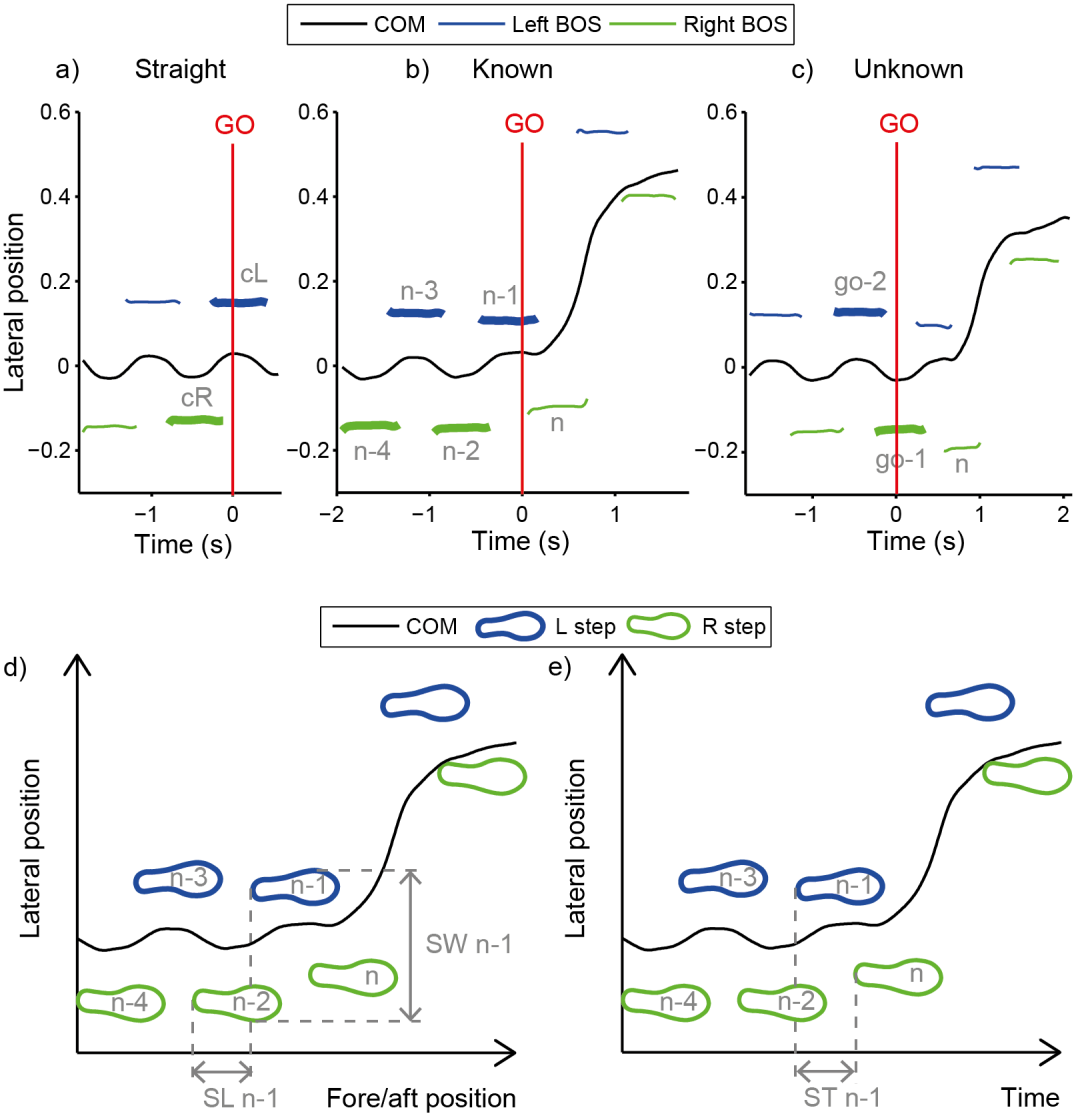
Step Analysis. Representative data from a single subject of center of mass (COM) and left and right base of support (BOS) position before and after the “GO” signal during each condition. **a)** Straight. “cL” and “cR” denote control left and right steps used for analysis. **b)** Known left. “n” denotes the maneuver step. The four steps prior to the n step, the n-1 through n-4 steps, were analyzed for specific anticipatory behavior. **c)** Unknown left. For this condition only, the “GO” signal denotes when subjects learned the maneuver direction. The two steps initiated prior to the “GO” signal, the go-1 and go-2 steps, were analyzed for general anticipatory behavior. The “n” or maneuver step is also shown for reference. **d)** Determination of step width (SW), and step length (SL) for the n-1 step of a Known left example trial. Step width for the n-1 step was calculated as the medio-lateral distance between the two 5^th^ Metatarsal markers at the n-1 initial contact. Step length for the n-1 step was calculated as the fore-aft distance between the two 5th Metatarsal markers at the n-1 initial contact. **e)** Determination of step time (ST). Step time for the n-1 step was calculated as the time between the n-1 and n step initial contact events.

We chose which steps to analyze for each of the conditions in order to capture specific and general anticipatory behaviors and to balance left and right limb sides. For the Straight condition, we analyzed the last left and right step—identified as the last two IC before the “GO” signal (Fig 2a)–to control for any potential gait adaptations made in response to viewing the visual cueing instructions. During the Known condition, specific anticipatory mechanisms can be identified by observing the gait adaptations subjects make during the steps leading up to the onset of an explicit maneuver that subjects are preparing to initiate at a known time and in a known direction. Thus, for the Known condition, we analyzed the four steps (called “n-1,” “n-2,” “n-3,” and “n-4”) before the maneuver step (“n” step) to examine specific anticipatory behavior (Fig 2b). The maneuver step was identified as the final IC before the visual feedback marker position moved outside of the baseline lane and was verified by visual inspection. The n-1 through n-4 steps were determined relative to the occurrence of the maneuver step, and thus would occur at variable times relative to the “GO” signal, depending on how quickly subjects initiated the maneuver. During the Unknown condition, general anticipatory mechanisms can be identified by observing the gait adaptations subjects make during the steps leading up to the “GO” signal. Because the maneuver direction is not known during this time, assuming that subjects were not employing a guessing strategy, gait adaptations should be made to facilitate maneuvers in multiple possible directions. Thus, for the Unknown condition, we analyzed the last two steps (called “go-1” and “go-2”)–identified as the last two IC before the “GO” signal (Fig 2c)–to investigate general anticipatory behaviors used to prepare for a maneuver that could be in more than one direction. The go-1 and go-2 steps were determined relative to the occurrence of the “GO” signal only and varied in timing relative to the maneuver initiation. Thus, the n-1 through n-4 steps that preceded the maneuver step, and the go-1 and go-2 steps that preceded the “GO” signal were time-linked to independent events and did not necessarily align in timing to each other. We analyzed more steps for the Known condition than the Unknown condition because this was necessary to identify temporal aspects of any specific anticipatory behaviors observed.

For every maneuver, we calculated the time and number of steps that occurred during the reaction period, defined by the instance the “GO” signal was presented until IC of the maneuver step. The number of steps initiated during the reaction period was identified from the number of IC events (including the maneuver step). If the maneuver step IC occurred before the “GO” signal, the values for time and number of steps were negative.

Minimum margin of stability was calculated in the following manner. First, COM lateral position was estimated as the midpoint of the lateral positions of the two Greater Trochanter markers. Next, XCOM position was calculated in the frontal plane using the following equation [17] based on the inverted pendulum model:

$$XCOM=COM+\text{​}COM\prime \sqrt{l/g}$$

XCOM = lateral extrapolated center of mass

COM = lateral center of mass position

COM’ = lateral center of mass velocity

*l* = pendulum length

g = gravitational constant

COM velocity was calculated as the derivative of COM position. Since the effective length of the inverted pendulum could change due to hip and knee flexion, “*l*” was defined as the instantaneous distance between the COM and the lateral Malleolus marker on the limb side that made the most recent initial contact.

Margin of stability was then calculated as the distance between the XCOM and the BOS of the limb that made the most recent IC. The BOS was estimated from the lateral position of the 5^th^ Metatarsal marker. Margin of stability had a positive sign when the XCOM was located medial of the BOS and negative when the XCOM was located lateral of the BOS. The minimum margin of stability was identified during stance phase for each limb side. We limited our examination of margin of stability to the frontal plane both because this was the primary plane in which the maneuvers were occurring, and because past research has suggested that human walking requires active control to maintain stability in the frontal plane [18–20].

We also examined step width, step length, and step time. Step width was calculated as the medio-lateral distance between the left and right 5^th^ Metatarsal markers at IC. Left step width was calculated at left IC (Fig 2d). Step length was calculated as the fore-aft distance between the left and right 5^th^ Metatarsal markers at IC. Left step length was calculated at left IC (Fig 2d). Left step time was calculated as the time between IC of the left foot and the following IC of the right foot (Fig 2e).

Finally, changes in stance phase limb flexion were examined during the analyzed steps. We identified the maximum stance phase hip and knee extension angles as measures of how “crouched” subjects’ posture were during stance. Knee extension angle during stance typically has two maxima—one occurring at IC and one occurring during mid/late stance. We examined only the maximum occurring at mid/late stance. Two subject’s hip angle data were excluded from analysis due to poor data capture. Joint angle convention was chosen such that extension was positive.

### Statistical Analysis

Due to non-normality of data, the non-parametric Wilcoxon rank-sum test was used to compare time and number of steps performed during the reaction period between Known and Unknown conditions. Significance was set at p < 0.05.

To investigate specific strategies used to prepare for maneuvers during the Known condition, we compared the control step to the four steps preceding the maneuver. We used separate two-way repeated measures ANOVAs (step by limb side) to test for differences in minimum margin of stability, step width, step length, step time, and stance phase maximum hip and knee extension angles. The two independent variables were “step,” which had five levels—control, n-4, n-3, n-2, and n-1 –and “limb side,” which had two levels—left and right. We did not assume left/right symmetry, and instead tested if limb side had a main or interaction effect on each metric. In the cases where limb side was found to have no effect, we collapsed data across sides. The decision to include limb side as a level was based on previous research that found left/right step width asymmetries during the approach step preceding turning [21] and left/right margin of stability asymmetries during walking [22,23]. If sphericity was violated, the Greenhouse-Geisser (G-G) F-statistic and p-value were used to test the main effects and interaction effect. When a significant main effect of step was found, Bonferroni-corrected pairwise comparisons were performed to look for differences between the control step and the other four steps. When a significant main effect of limb side was found, a t-test was performed to compare the left and right. When a significant interaction of step and limb side was found, simple effects analysis was conducted (i.e. a t-test compared limb side at each step). Significance was set at the p < 0.05 level for the repeated measures ANOVAs, pairwise comparisons, and t-tests.

To investigate general strategies subjects used to facilitate maneuverability for the Unknown condition, we compared the control step to the two steps preceding the “GO” signal (go-2 and go-1 step). Again, separate two-way repeated measures ANOVA (step by limb side) were performed to test for differences in the same six metrics examined during the Known condition. The three levels of step were control, go-2, and go-1. The post-hoc tests to follow up a significant main effect were the same as for the Known condition.

## Results

### Reaction Period

The reaction period between the “GO” signal and the IC of the maneuver step was significantly shorter (p < 0.001) and required less steps (p < 0.001) for the Known condition than for the Unknown condition. For the Known condition, subjects initiated the maneuver step (but kept their COM within the baseline lane) almost immediately **before** the “GO” signal, as reflected in the negative values of (mean ± std. dev.) -0.060 ± 0.566 steps and -0.007 ± 0.132 s. For the Unknown condition, subjects required 1.910 ± 0.221 steps (including the maneuver step) and 0.601 ± .078 seconds **after** the “GO” signal to initiate the maneuver.

### Known Condition

For the Known condition, all metrics examined—margin of stability, step width, step length, step time, maximum stance phase hip extension angle, and maximum stance phase knee extension angle—had a significant main effect of step comparing the four pre-maneuver steps and the control step (p < 0.05) (see Table 1 for means and standard deviations of all variables and Table 2 for associated statistical analyses). For the Known condition, none of the metrics had a significant main effect of limb side (p > 0.05) (Tables 1 and 2). Thus, to compare between steps, the data was collapsed across limb side for each metric.

**Table 1:** Means and standard deviations for Known condition.

METRIC	EFFECT	LEVEL	SUB-LEVEL^1^	MEAN	STD. DEV.
Margin of stability	**step**	control		0.067	0.007
		**n-4**		**0.074**	**0.01**
		**n-3**		**0.075**	**0.013**
		**n-2**		**0.085**	**0.018**
		**n-1**		**0.021**	**0.032**
	step*side	**control**	***left***	**0.073**	**0.012**
			***right***	**0.061**	**0.01**
		**n-4**	***left***	**0.08**	**0.012**
			***right***	**0.068**	**0.012**
		**n-3**	***left***	**0.081**	**0.017**
			***right***	**0.069**	**0.014**
		**n-2**	***left***	**0.089**	**0.02**
			***right***	**0.08**	**0.019**
		n-1	left	0.015	0.048
			right	0.027	0.028
Step width	**step**	control	—	0.242	0.029
		n-4	—	0.252	0.025
		n-3	—	0.252	0.028
		**n-2**	—	**0.261**	**0.031**
		n-1	—	0.248	0.029
Step length	**step**	control	—	0.661	0.044
		n-4	—	0.638	0.049
		n-3	—	0.624	0.061
		n-2	—	0.581	0.121
		n-1	—	0.561	0.134
Step time	**step**	control	—	0.552	0.04
		n-4	—	0.532	0.047
		n-3	—	0.512	0.065
		n-2	—	0.49	0.094
		**n-1**	—	**0.456**	**0.094**
Maximum hip extension angle	**step**	control	—	9.518	6.14
		n-4	—	5.959	7.119
		n-3	—	3.668	9.4
		n-2	—	2.366	10.904
		**n-1**	—	**-3.206**	**12.845**
Maximum knee extension angle	**step**	control	—	-8.17	3.413
		n-4	—	-11.09	4.835
		n-3	—	-12.238	6.198
		n-2	—	-14.21	8.385
		**n-1**	—	**-18.964**	**10.593**

^1^The sublevel of limb side is shown only for Margin of Stability since this was the only metric for which there was a significant interaction effect of step and limb side.

Data from all 14 subjects were used for each metric except hip angle, where only 12 subjects were used. Values are presented for significant main and interaction effects only. Means significantly different from the control step are in bold. Significantly different pairs within step (simple effects analysis) are in italics.

**Table 2:** Known target direction statistical analysis.

METRIC	MAIN EFFECT	NUM. DF	DENOM. DF	F	P-VALUE	SIG. DIFF. PAIRS^1^	P-VALUE	SIMPLE EFFECTS OF SIDE W/IN STEP	P-VALUE
Margin of stability	**step**	1.34	17.424	33.505	**<0.001**	**n-4 > control**	**0.007**	—	—
						**n-3 > control**	**0.004**	—	—
						**n-2 > control**	**0.001**	—	—
						**n-1 < control**	**<0.001**	—	—
	side	1	13	1.988	0.182	—	—	—	—
	step*side	1.173	15.254	4.767	**0.04**	—	—	***control*: *L > R***	***0*.*015***
						—	—	***n-4*: *L > R***	***0*.*007***
						—	—	***n-3*: *L > R***	***0*.*026***
						—	—	***n-2*: *L > R***	***0*.*025***
Step width	**step**	4	52	7.729	**<0.001**	**n-2 > control**	**0.003**	—	—
	side	1	13	2.719	0.123	—	—	—	—
	step*side	1.897	24.659	0.18	0.826	—	—	—	—
Step length	**step**	1.129	14.671	7.538	**0.013**	none	—	—	—
	side	1	13	0.24	0.632	—	—	—	—
	step*side	2.388	31.047	0.239	0.826	—	—	—	—
Step time	**step**	1.36	17.675	12.845	**0.001**	**n-1 < control**	**0.01**	—	—
	side	1	13	3.882	0.07	—	—	—	—
	step*side	1.906	24.78	0.955	0.395	—	—	—	—
Max. hip ext. angle	**step**	1.426	15.682	10.801	**0.002**	**n-1 < control**	**0.028**	—	—
	side	1	11	3.204	0.101	—	—	—	—
	step*side	2.16	23.763	2.18	0.132	—	—	—	—
Max. knee ext. angle	**step**	1.207	15.686	11.545	**0.003**	**n-1 < control**	**0.028**	—	—
	side	1	13	1.241	0.286	—	—	—	—
	step*side	1.876	24.392	1.413	0.262	—	—	—	—

^1^For the follow-up to a significant main effect of step, only comparisons vs. the control step were considered. Means significantly different from the control step are in bold. Significantly different pairs within step (simple effects analysis) are in italics.

**Fig 3:**
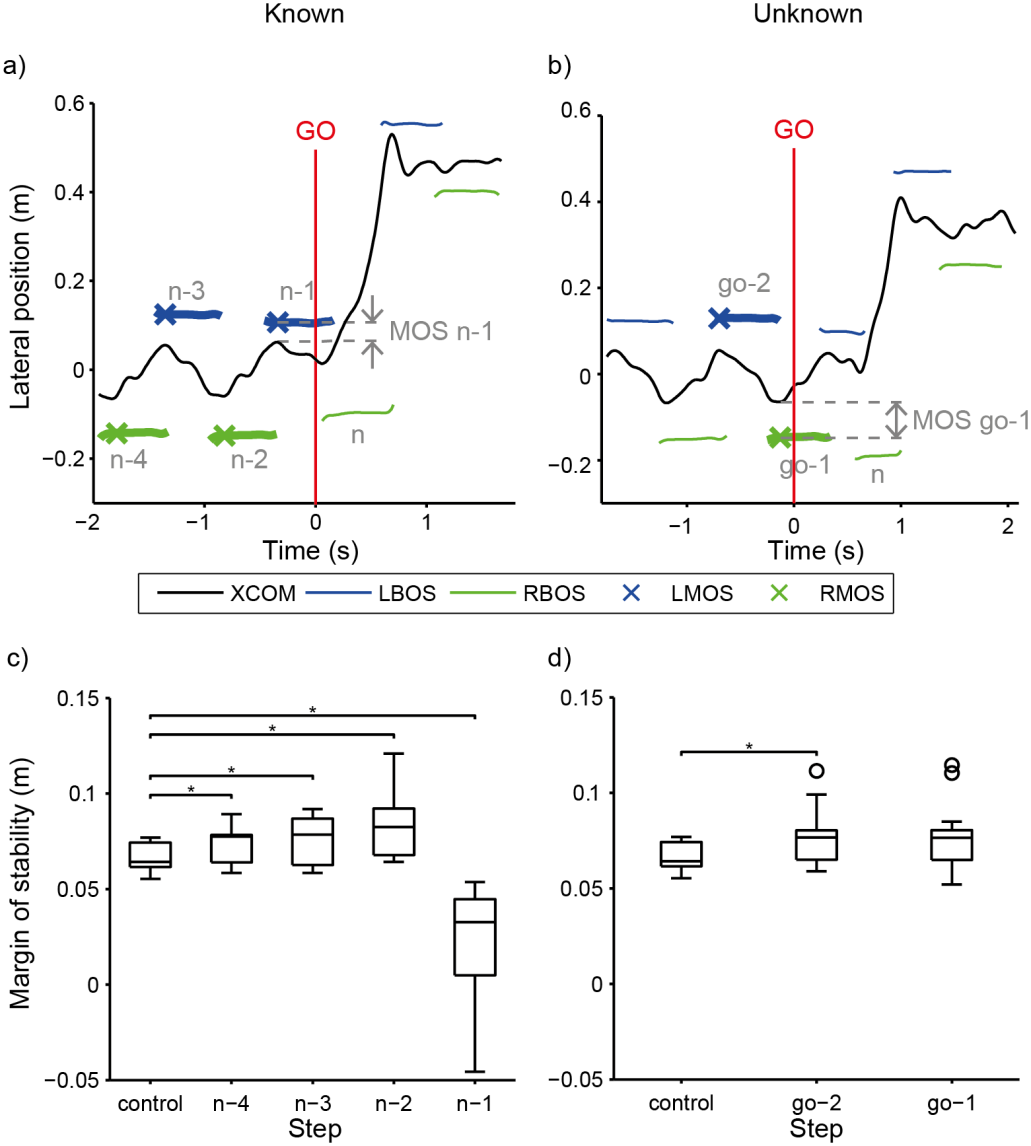
Minimum Margin of stability. Example margin of stability data for one subject during **a)** a Known left trial and **b)** an Unknown left trial. Left (L) and Right (R) minimum margin of stability (MOS), denoted by “x” marker at each step, is the minimum distance between the base of support (BOS) and extrapolated center of mass (XCOM). **c)** Minimum margin of stability, Known condition. The minimum margin of stability was significantly larger for the n-4 through n-2 steps, and significantly smaller one step prior to the maneuver, the n-1 step (ipsilateral to the target), compared to the control step. **d)** Minimum margin of stability, Unknown condition. Minimum margin of stability was significantly larger 2 steps before the “GO” signal, the go-2 step. (*)Step is significantly different (p< 0.05) from control. For **c)** and **d)** the Box plots are collapsed across limb side to show main effect of step. Each box represents 14 data subjects’ data.

Pairwise comparisons found that the minimum lateral margin of stability for all four of the pre-maneuver steps analyzed were significantly different from the control step (p < 0.05) (Table 2). Minimum margin of stability was larger during the n-4, n-3, and n-2 steps (10, 13, and 27%, respectively) than during the control step (Table 1 and Fig 3a and 3c). During the n-1 step, minimum margin of stability was 69% smaller (on the limb side ipsilateral to the target direction) than during the control step (Table 1 and Fig 3a and 3c). Minimum margin of stability also showed a significant interaction effect between step and limb side (p < 0.05). The results of the simple effects analysis found that the minimum margin of stability was larger on the left side vs. the right side for the control, n-4, n-3, and n-2 steps (p < 0.05) (Table 2). No difference between left and right sides was found for the n-1 step (p > 0.05).

**Fig 4:**
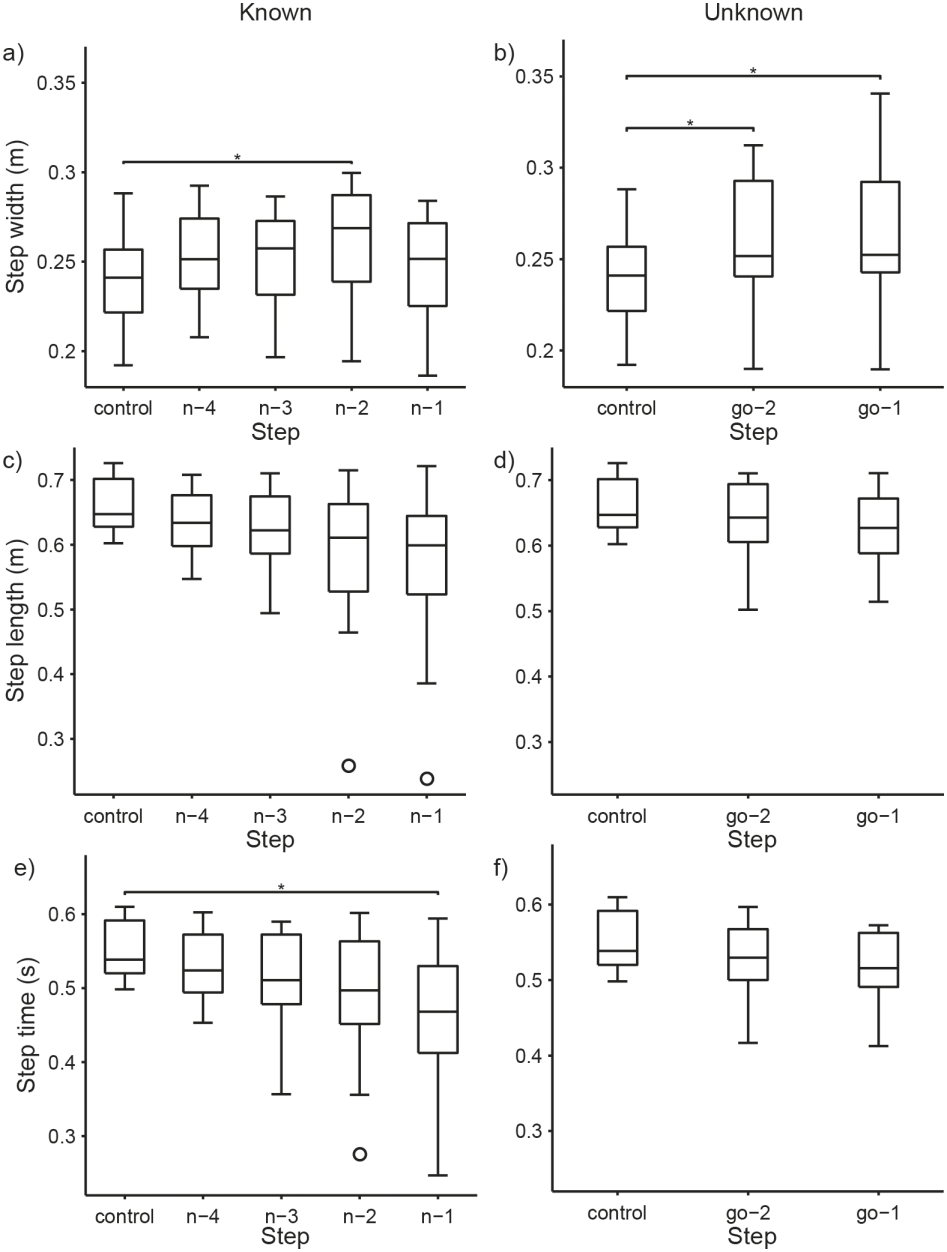
Step Width, Step Length, and Step Time. Each box plot is collapsed across limb side to show main effect of step on step width, step length and step time. Each box represents 14 subjects’ data. **a)** Step width, Known condition. The n-2 step width was significantly larger than the control step (contralateral to the target). **b)** Step width, Unknown condition. Step width was significantly increased for both steps immediately preceding the “GO” signal compared to the control step. **c)** Step length, Known condition. There was a significant effect of step on step length, but no pairwise comparisons vs. the control step were significant. **d)** Step length, Unknown condition. There were no significant effects of step on step length. **e)** Step time, Known condition. Compared to the control step, step time was significantly shorter during the n-1 step. **f)** Step time, Unknown condition. There was a significant effect of step on step time, but neither the go-2 or go-1 steps were significantly different from the control step. (*)Step is significantly different (p<0.05) from control.

For step width, pairwise comparisons found that only the n-2 step was significantly different than the control step (p < 0.05) (Table 2). The n-2 step width was 8% wider than the control step (Table 1 and Fig 4a).

Although a significant main effect of step was found for step length, no pairwise comparisons found significant differences between the control step and any of the pre-maneuver steps (p > 0.05) (Table 2 and Fig 4c).

**Fig 5:**
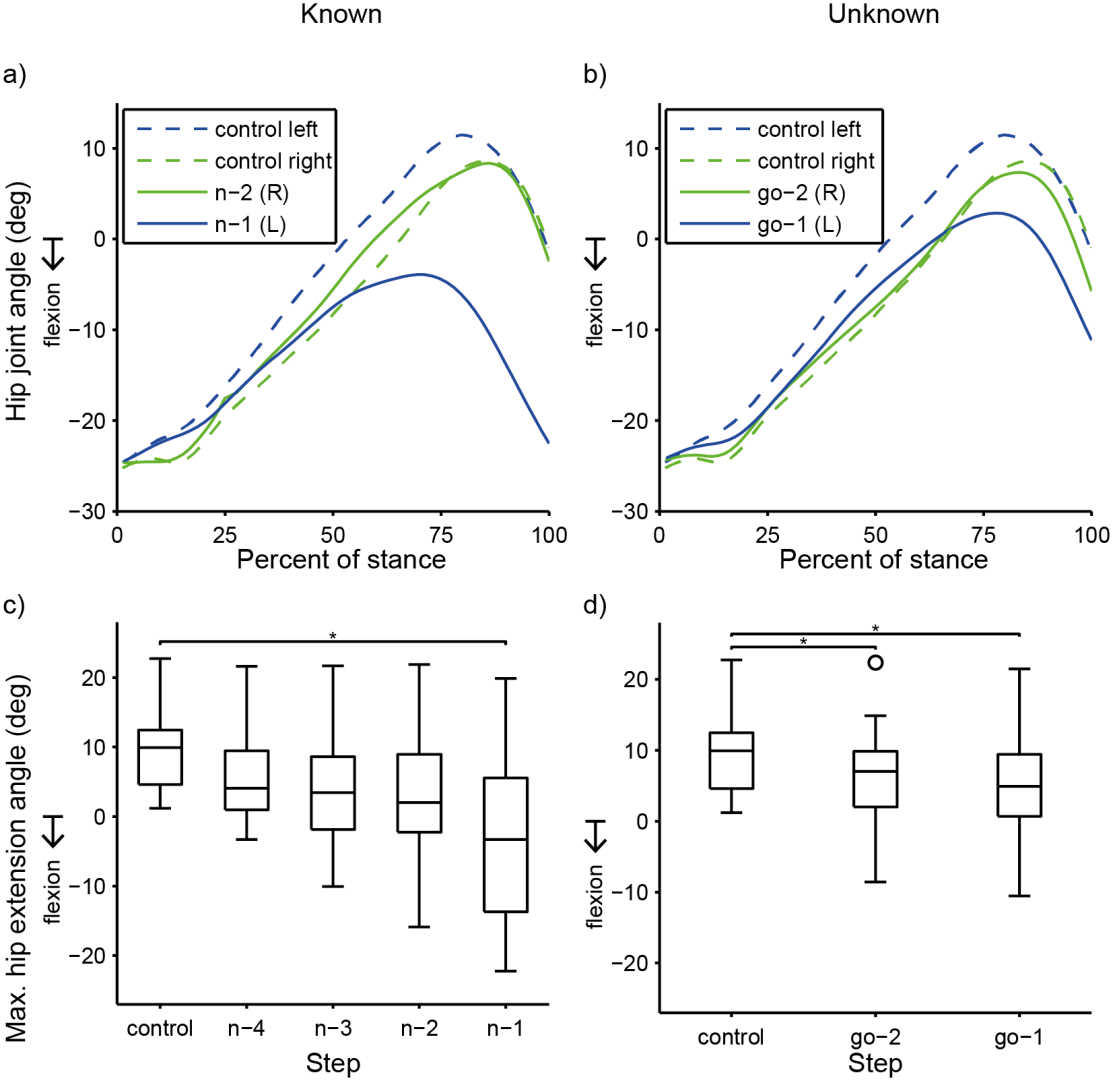
Hip Joint Angle. Example hip joint angle data for one subject comparing a Straight trial vs. **a)** a Known left trial and **b)** an Unknown trial. **c, d)** Each box plot is collapsed across limb side to show main effect of step on peak stance phase hip extension angle. Each box represents 12 subjects’ data (two subjects excluded). **c)** Known. The hip was significantly more flexed at the n-1 step compared to the control step. **d)** Unknown. The hip was significantly more flexed at both steps immediately preceding the “GO” signal compared to the control step. (*)Step is significantly different (p<0.05) from control.

**Fig 6:**
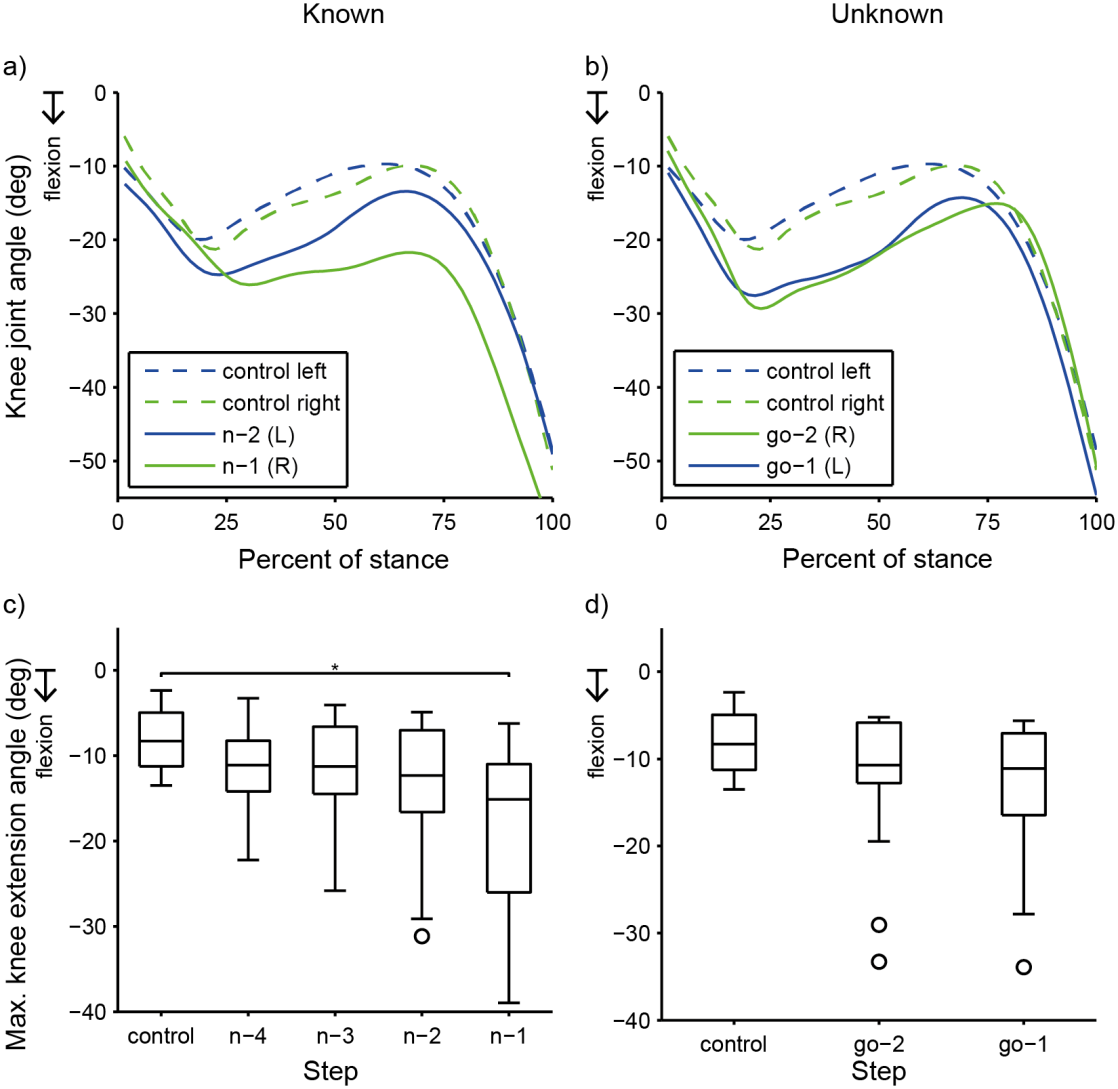
Knee Joint Angle. Example knee joint angle data for one subject comparing a Straight trial vs. **a)** a Known right trial and **b)** an Unknown trial. **c, d)** Each box plot is collapsed across limb side to show main effect of step on peak stance phase knee extension angle. Each box represents 14 subjects’ data. **c)** Known. The knee was significantly more flexed at the n-1 step compared to the control step. **d)** Unknown. There was a significant effect of step on peak knee extension angle, but pairwise comparisons vs. the control step were not significant. (*)Step is significantly different (p<0.05) from control.

For step time, stance phase maximum hip extension angle, and stance phase maximum knee extension angles, pairwise comparisons found significant differences only between the n-1 step and the control step (p < 0.05) (Table 2). Compared to the control step, during the n-1 step, step time was 17% shorter (Table 1 and Fig 4e), and the hip and knee were significantly more flexed (Table 1 and Figs 5a and 5c and 6a and 6c).

### Unknown Condition

For the Unknown condition, all the variables we examined, except step length, had a significant main effect of step when comparing the two pre-“GO” signal steps vs. the control step (p < 0.05) (see Table 3 for means and standard deviations of all variables and Table 4 for associated statistical analyses). No significant main effect of limb side or interaction between step and side was observed for any of the metrics (p > 0.05) (Table 4), with the exception of minimum margin of stability, which had a significant main effect of limb side (p < 0.05).

**Table 3:** Means and standard deviations for Unknown condition.

METRIC	EFFECT	LEVEL	MEAN	STD. DEV.
Margin of stability	**step**	control	0.067	0.007
		**go-2**	**0.077**	**0.015**
		go-1	0.078	0.017
	**side**	left	0.079	0.006
		right	0.068	0.006
Step width	**step**	control	0.242	0.029
		**go-2**	**0.261**	**0.036**
		**go-1**	**0.265**	**0.039**
Step length	step	control	0.661	0.045
		go-2	0.641	0.058
		go-1	0.630	0.055
Step time	**step**	control	0.552	0.040
		**go-2**	**0.530**	**0.049**
		**go-1**	**0.519**	**0.044**
Maximum hip extension angle	**step**	control	9.518	6.140
		**go-2**	**6.521**	**7.845**
		**go-1**	**5.100**	**8.475**
Maximum knee extension angle	**step**	control	-8.170	3.413
		go-2	-12.560	8.834
		go-1	-13.270	8.774

Data from all 14 subjects were used for each metric except hip angle, where only 12 subjects were used. Values are presented for significant main and interaction effects only, except for the case of step length, where there were no significant effects, but we have provided means for comparison. Means significantly different from the control step are in bold.

**Table 4:** Unknown target direction statistical analysis.

METRIC	MAIN EFFECT	NUM. DF	DENOM. DF	F	P-VALUE	SIG. DIFF. PAIRS^1^	P-VALUE
Margin of stability	**step**	**1.132**	**14.71**	**7.381**	**0.014**	**go-2 > control**	**0.039**
	**side**	**1**	**13**	**7.854**	**0.015**	**L > R**	**0.015**
	step*side	2	26	0.816	0.453	**—**	**—**
Step width	**step**	**1.193**	**15.51**	**12.82**	**0.002**	**go-2 > control**	**0.007**
						**go-1 > control**	**0.009**
	side	1	13	0.101	0.756	**—**	**—**
	step*side	2	26	0.332	0.72	**—**	**—**
Step length	step	1.183	15.384	3.563	0.073	**—**	**—**
	side	1	13	0.069	0.797	**—**	**—**
	step*side	2	26	1.317	0.285	**—**	**—**
Step time	**step**	**1.089**	**14.15**	**5.069**	**0.038**	**—**	**—**
	side	1	13	1.638	0.223	**—**	**—**
	step*side	1.222	15.887	0.438	0.557	**—**	**—**
Maximum hip extension angle	**step**	**1.08**	**11.89**	**9.44**	**0.009**	**go-2 vs. control**	**0.047**
						**go-1 vs. control**	**0.026**
	side	1	11	0.561	0.469	**—**	**—**
	step*side	1.025	11.28	0.218	0.656	**—**	**—**
Maximum knee extension angle	**step**	**1.047**	**13.61**	**6.104**	**0.026**	**—**	**—**
	side	1	13	0.289	0.6	**—**	**—**
	step*side	1.149	14.942	2.392	0.141	**—**	**—**

^1^For the follow-up to a significant main effect of step, only comparisons vs. the control step were considered. Means significantly different from the control step are in bold.

Pairwise comparisons found that the minimum margin of stability at the go-2 step was significantly (15%) larger than the control step (p < 0.05) (Tables 3, 4 and Fig 3b and 3d). The minimum margin of stability during the go-1 step was not different from the control step (p > 0.05). Minimum margin of stability also had a significant main effect of limb side: the left margin of stability was significantly 16%) larger than the right margin of stability (Table 3).

Pairwise comparisons found that step width for both pre-“GO” signal steps were significantly different from the control step (p < 0.05) (Table 4). The go-2 and go-1 step width were 8% and 9% wider, respectively, than the control step width (Table 3 and Fig 4b).

For the Unknown condition, step length had no significant main effect for step (p > 0.05) (Table 4 and Fig 4d). Similarly, pairwise comparisons found no significant differences in step time (p > 0.05) (Table 4 and Fig 4f) when comparing the pre-“GO” signal steps to the control step.

Pairwise comparisons found that the hip joint angle was significantly less extended (more flexed) during both the go-2 step and go-1 step than during the control step (p < 0.05) (Table 4 and Fig 5b and 5d). Pairwise comparisons found no significant differences in knee angle between either of the pre-“GO” signal steps and the control step (p < 0.05) (Table 4 and Fig 6b and 6d).

Individual subjects’ data for all metrics can be found in S1 Table.

## Discussion

To investigate specific and general strategies used to facilitate locomotor maneuvers, we manipulated subjects’ ability to anticipate the direction of an upcoming lateral change of direction.

To examine specific anticipatory adjustments, we observed the four steps immediately preceding a maneuver that was to be performed at a known time in a known direction. We hypothesized that to facilitate a specific change of direction, subjects would modulate passive stability via proactive direction-specific decreases in margin of stability. Our results support this hypothesis: subjects significantly decreased lateral margin of stability on the side ipsilateral to the maneuver during the step immediately preceding the maneuver. Our results confirmed that the anticipatory decrease in lateral margin of stability was specific to the n-1 step only.

To examine general anticipatory adjustments, we observed the two steps immediately preceding the instant when subjects received information about the direction of the maneuver (i.e. the two steps prior to the “GO” signal). When the direction of the maneuver was unknown, we hypothesized that subjects would make general anticipatory adjustments that would improve their ability to actively initiate a maneuver in multiple directions via increases in step frequency, step width, and stance limb flexion. This second hypothesis was partially supported: subjects increased step width and stance phase hip flexion during the two steps immediately prior to the “GO” signal, but significant differences were not observed in step frequency, knee flexion, or step length.

### Known Maneuver Conditions

When subjects could anticipate the time and direction of an upcoming lateral maneuver, they made several preparatory locomotor adaptations prior to executing a side-step. As early as four steps prior to the maneuver, subjects increased their lateral margin of stability bilaterally (the n-4 and n-3 steps). This increase in margin of stability was likely due to changes in the center of mass dynamics as step width was not significantly different from the control step during the n-4 and n-3 steps. Then, two steps prior to the maneuver (the n-2 step), subjects changed their foot placement, taking a wider step in the direction opposite of the impending maneuver. This “step out” contributed to an increased lateral margin of stability contralateral to the maneuver direction, and likely increased the frontal plane gravitational moments about the hip and subtalar joints that would passively accelerate the COM laterally [18] towards the target lane. One step prior to the maneuver (the n-1 step), step time decreased, but step width and length were not significantly different from the control step. Due to changes in COM dynamics, subjects’ XCOM position was significantly closer to their lateral BOS on the side ipsilateral to the target during this final pre-maneuver step. During the n-1 step, subjects also assumed a more crouched posture, increasing both stance phase hip and knee flexion of the inside limb.

As subjects then transitioned to the actual maneuver, they made a short and rapid step to place the outside limb in contact with the ground. Subjects timed this foot contact to coincide very closely with the “GO” signal—IC of the maneuver step (on the side contralateral to maneuver direction) occurred, on average, 7 milliseconds prior to the “GO” signal. Subjects then executed a side-step maneuver by pushing off of the outside limb and making a swift lateral step with the inside limb to the target lane. Maneuver style—side-step or cross-over step—will likely impact passive stability. In the current study, we analyzed only side-step maneuvers. While this was the most frequent maneuver style, subjects did at times choose a cross-over step maneuver, in particular when they were becoming familiar with the task. The COM is always within the BOS for a side-step [7], while, in contrast, the COM crosses over the supporting limb and moves outside of the BOS for a cross-over step. One reason subjects may have preferred the side-step is that it allowed them greater passive stability during the execution of the maneuver.

A simple inverted pendulum model predicts that reductions in margin of stability should result in a proportional decrease in the lateral impulse required to move one’s XCOM beyond one’s BOS [17]. The model predicts that applied impulses that do not result in the XCOM moving beyond the BOS will passively self-stabilize. In the current experiment, subjects decreased their minimum lateral margin of stability by 69% ipsilateral to the maneuver during the final pre-maneuver step. When preparing to maneuver, this decrease in margin of stability will decrease the volitional force threshold required to move the XCOM beyond the BOS. That subjects chose to manipulate an aspect of passive stability prior to initiating a maneuver is consistent with past literature demonstrating that people lean into a turn in preparation for the turn [6]. One potential limitation of the current research was that whole body lateral COM location was estimated from pelvis markers. This method might underestimate actual changes in margin of stability by failing to capture effects of trunk lean.

Manipulating passive stability could be motivated by a combination of factors that make this strategy beneficial when the time and direction of the maneuver are predictable. First, assuming that the postural change associated with reducing margin of stability does not impact the ability to actively generate laterally-directed forces, this gait adaptation should reduce the metabolic cost of a specific maneuver by requiring a smaller volitional impulse to move laterally. Second, reducing passive stability could simplify the neural control of maneuvering. Evidence from passive dynamic walking robots suggests that many motions occurring during gait can be produced without active control [24]. Thus, positioning the body to take advantage of passive mechanics could simplify control of maneuver execution. Finally, the central nervous system may use anticipatory postural adjustments to maintain equilibrium during the subsequent rapid movements of the actual maneuver [11,25]. During the maneuver the BOS moves to a new location. Preemptively leaning in the direction of the future maneuver places the XCOM closer to a position that will be passively stable (within the future BOS) during and after the maneuver. In the current study, decreasing margin of stability in the direction of the maneuver might have minimized the postural disturbance that would be created by the impending maneuver. Similarly, it has been observed that specific anticipatory adjustments made in COM and center of pressure location are tailored to the specific demands of an upcoming turn, including variables of gait speed and turn angle [6].

There are tradeoffs to making anticipatory decreases in margin of stability. Most notably, an individual becomes more susceptible to the effects of perturbations. While a smaller margin of stability has not been shown to increase the risk of falling [26], it does imply that a smaller magnitude perturbation is needed to move the XCOM outside of the BOS, a situation where active correction is required to prevent a fall. Even if a perturbation does not cause a fall, decreasing the margin of stability increases the likelihood that a perturbation may cause an undesirable change in direction.

However, these risks can be minimized when the maneuver direction is known. In the current study, subjects exhibited at least three actions that may have limited the risks associated with perturbations during the period of reduced margin of stability. First, the decrease in margin of stability during the final step before the maneuver (n-1 step) was coupled with a 17% decrease in step time (time from n-1 IC until the maneuver initiation step IC). The shorter step time decreased the duration of potential exposure to lateral perturbations while less passively stable. While subjects may have actively selected a shorter step time for the n-1 step, it is also possible that the observed decrease in step time could have been a direct result of only analyzing the fastest maneuvers. Second, subjects may have minimized risk by limiting decreases in margin of stability spatially to only the intended direction of travel and temporally to only the n-1 step. During the n-2 and n-4 steps, which occurred on the contralateral direction, and the n-3 step, which occurred on the ipsilateral direction, margin of stability actually increased compared to the control step. Finally, subjects may have also used an active strategy to offset some of the risk by increasing hip and knee flexion during the n-1 step. A crouched posture may improve stability by positioning the limb so that it can either shorten or lengthen in response to a perturbation [27]. The flexed position of the inside limb during the n-1 step may have also improved the subjects’ active ability to rapidly unload the limb at the moment the outside limb contacted the ground and initiated the maneuver.

### Unknown condition

When subjects knew the time but not the direction of a maneuver, they had different preparatory strategies than during the Known condition. During the Unknown condition, subjects made kinematic changes during the two steps initiated prior to the “GO” signal, the instant when the maneuver direction was revealed. Subjects significantly increased step width and stance-phase hip flexion during the two pre-“GO” signal steps. They also significantly increased lateral margin of stability during the go-2 step compared to the control step. Significant differences were not observed between the control step and the pre-“GO” signal steps for step time, step length, or stance-phase knee flexion.

Unlike the Known condition, despite prior knowledge of the timing of the maneuver, during the Unknown condition subjects did not initiate the maneuver at the “GO” signal. From the “GO” signal, subjects required an additional two steps to initiate the maneuver. Previous work has observed that time is required to prepare for a maneuver [7,28], and that changes occur in step width, trunk orientation, and frontal plane hip and knee moments during the immediate steps leading up to and including the maneuver step as the preparation time between maneuver instructions and the actual maneuver decreases [7,29,30]. Our goal with the Unknown condition was not to further examine the specific strategies used for initiating an unanticipated maneuver once movement direction is known, but rather to examine general preparation strategies used to prepare for possible maneuvers in multiple directions.

General anticipatory strategies used to facilitate a possible rapid maneuver in multiple directions should address factors that allow the change of direction to be performed in an effective and safe manner. These factors include the ability to quickly position the limbs to generate the necessary impulse required for the maneuver and positioning the body to counteract the destabilizing effects of initiating a rapid change of direction. Both preemptive positioning of the limbs to better generate forces in multiple directions and increasing the speed at which this posture can be achieved should result in a more rapid production of the translational and/or rotational forces about the center of mass necessary for the maneuver.

In the current study, subjects assumed a more crouched posture during the go-1 and go-2 steps, significantly increasing stance-phase hip flexion. Flexing the hip and knee joints at mid-stance should help prepare for movement in multiple directions, because the limb is in a position where it is able to effectively shorten or lengthen as needed. The tradeoff to this strategy is that a flexed stance limb increases stress placed on muscles and bones [31] and will increase the metabolic cost of transport [15].

Subjects took wider steps prior to the “GO” signal, which may have assisted with both force production and stability of the maneuver. Taking wider steps places the line of action of the sagittal plane joint extensor muscles in an improved position to generate a lateral impulse to accelerate the body in the frontal plane toward the midline, and therefore might improve individuals’ ability to initiate lateral movements. In addition, choosing wider steps tends to increase margin of stability bilaterally, which could help offset future destabilizing perturbations inherent to initiating a rapid maneuver. Our results are consistent with previous research that has demonstrated increased step width during gait in preparation for a rapid gait adaptation task [13,32]. However, taking wider steps increases metabolic cost [14] and its role for enhancing the ability to rapidly initiate a maneuver is not entirely clear. If an increased step width could independently improve maneuver performance, we would have expected to observe wider steps during conditions when subjects could fully plan for a maneuver. That we did not observe an increase in step width during the n-1 step of the Known condition suggests that the value of increasing step width may be situation dependent.

Alternatively, to facilitate bilateral maneuverability, individuals could have decreased lateral margins of stability bilaterally. Subjects in the current study did not choose this strategy. Even though the environment, treadmill walking, was highly predictable and the chance of receiving an external perturbation was small, decreasing margin of stability in multiple directions may have been a “risky” behavior. In this situation, the risk would not have been falling, but rather an increased probability of an undesirable acceleration of the COM. Success in the prescribed task required the ability to initiate multi-directional maneuvers as quickly as possible, so any acceleration directed away from the future maneuver would limit performance. In addition, decreasing margin of stability by decreasing step width may also limit the ability to generate sufficient lateral impulses required for initiating and executing the maneuver.

To increase the speed of positioning the limbs in a posture advantageous for force generation, subjects could have adopted a general strategy of decreasing step length and step time. In the current study, it took subjects two steps, on average, to initiate the maneuver step after the “GO” signal. If multiple steps are required to appropriately position oneself to execute the maneuver, then increasing step frequency could result in a faster maneuver initiation. While we observed a significant main effect of “step” on step time, the post-hoc comparisons between steps were not significant (control vs. go-1 step t-test: p = 0.052), likely due to a lack of statistical power. Alternatively, increasing step frequency might be avoided because of the associated increase in metabolic cost [16]. Also, it has been hypothesized that increasing step frequency during a gait adaptation task could be undesirable because increased movement speeds might decrease movement accuracy [13].

There are some limitations to the methods we used to evaluate general preparatory strategies during the Unknown Condition. First, the analysis of the go-1 step to characterize “general” preparations made in anticipation of an unknown maneuver may be limited. As a result of our definition, the “GO” signal always occurred during the go-1 step (0.273 ± 0.042 seconds after the go-1 step’s initial contact). As such, the behaviors observed during the go-1 step could have been influenced by specific changes made mid-step once the movement direction was revealed. Second, subjects could have used a guessing strategy that involved anticipatory adjustments prior to the “GO” signal. This strategy could have improved their maneuver performance when the direction was guessed correctly as opposed to waiting until the “GO” signal was presented to begin to initiate the maneuver in a given direction. While we cannot rule out this possibility, the best indication that subjects were not guessing is that, on average, subjects took approximately 2 steps and over half a second after the “GO” signal was presented to initiate the maneuver during the Unknown condition. If subjects had anticipated and guessed the direction of travel during Unknown trials, we would expect that the reaction period would be similar to the values observed during the Known condition, where the maneuver was initiated almost immediately at the “GO” signal.

### Margin of stability asymmetry

People may choose to adapt frontal plane asymmetries as a general strategy for increasing passive stability. Of the metrics we examined, only margin of stability demonstrated an effect of limb side. Specifically, margin of stability showed a significant interaction effect between limb side and step for the Known condition and a main effect of limb side for the Unknown condition. The significant interaction in the Known condition was due to a larger minimum margin of stability on the left vs. right side for all steps except the n-1 step. In the Unknown condition, left minimum margin of stability was larger than right across all steps. Asymmetries in frontal plane margins of stability have been observed in both control [22,23] and above-knee amputee populations [33] with a greater margin of stability maintained on the impaired side. It has been suggested that during normal gait, lower limb asymmetries could be attributed to differences in the functional tasks performed by each limb [34] with the left limb controlling medio-lateral balance [35] and the right limb being associated with propulsion [36]. Consistent with these observations, the greater margins of stability on the left side found in the current study may have been a general strategy individuals selected to improve the ability of the limb to control frontal plane stability. Interestingly, during the n-1 step of the Known condition, there was no effect of limb side observed. This supports the idea that subjects adopted a specific strategy of reducing passive stability during the final pre-maneuver step in order to facilitate the upcoming maneuver. During the n-1 step, controlling stability through passive mechanisms may not have been a priority and, as a result, asymmetry in frontal plane margins of stability may not have been present during this step.

### Clinical implications

Stability is challenged during walking maneuvers [2] that require ongoing postural adjustments and base of support corrections to ensure safe redirection of the momentum of the trunk and limbs. Although changing direction is a frequent occurrence during community ambulation [37,38], the performance of safe and efficient maneuvers is often challenging for individuals with sensory-motor deficits. During straight-ahead walking, to compensate for sensory and motor deficits that are known to impair balance [39,40], individuals with neurologic injury often depend heavily on general, passive mechanisms to provide locomotor stability (e.g. decreasing walking speed [41,42], increasing step width [14,43–45], and increasing double support time [46,47]). Older adults [48,49] and individuals with neurologic injury [50,51] may also select compensatory stepping patterns during maneuvers to enhance their passive stability (e.g. increasing lateral margins of stability and choosing slow, multi-step turns). If an individual has difficultly sensing and/or responding to specific perturbations, adopting general, passive mechanisms of stability could be advantageous because this strategy decreases the ongoing demands placed on the central nervous system to actively maintain stability. In the current study, we observed that healthy control subjects decreased their lateral margin of stability in preparation for an upcoming maneuver. This strategy likely improved the maneuver’s efficiency. In contrast, for individuals’ with sensory-motor impairments, reliance on general, passive stabilization strategies or an inability to safely modulate their reliance on such strategies, may contribute to inefficient maneuvers and/or may increase fall risk in situations requiring greater dependence on active mechanisms of stability, such as during rapid or unanticipated maneuvers. To improve community ambulation of those with sensory-motor deficits, becoming proficient at making specific responses to perturbations may be a skill that will allow individuals to safely decrease their reliance on energetically-costly general, passive mechanisms of stability in favor of more efficient but sensory-motor demanding active mechanisms of stability.

## Conclusions

Subjects made anticipatory adjustments to prepare for the initiation of a maneuver based on the information available about the upcoming maneuver. In the current study, when subjects could predict the time and direction of a lateral “lane change” maneuver, they adapted their gait in preparation for a specific maneuver during the steps immediately preceding maneuver initiation. In particular, margin of stability decreased in the direction of the anticipated maneuver, which might have taken advantage of the body’s passive dynamics to increase energetic efficiency and to simplify the neural control required for the maneuver. Subjects may have minimized the potential risks associated with this specific anticipatory strategy by limiting the duration of the reduced margin of stability to only the step immediately preceding the maneuver and restricting the decrease in margin of stability to only the intended direction of travel. When subjects were not able to predict the direction of an upcoming maneuver, they made general gait adaptations that prepared them to move in multiple directions: increasing step width and assuming a more crouched posture during stance phase. These adaptations likely improved the ability to rapidly produce forces in multiple directions and to maintain the body’s equilibrium during the onset and execution of a rapid maneuver. However these general anticipatory strategies likely increased energetic cost. Overall, the anticipatory strategies employed to prepare for a maneuver appear highly influenced by knowledge of the timing and direction of an upcoming maneuver.

## Supporting Information

S1 Table Individual subjects’ data for demographics and metrics. (XLSX)
